# Characterization and genomic analysis of chromate resistant and reducing *Bacillus cereus *strain SJ1

**DOI:** 10.1186/1471-2180-10-221

**Published:** 2010-08-19

**Authors:** Minyan He, Xiangyang Li, Liang Guo, Susan J Miller, Christopher Rensing, Gejiao Wang

**Affiliations:** 1 State Key Laboratory of Agricultural Microbiology, College of Life Science and Technology, Huazhong Agricultural University, Wuhan, 430070, China; 2 Department of Soil, Water and Environmental Science, The University of Arizona, Tucson, AZ 85721, USA; 3 Biotechnology Computing Facility, Arizona Research Laboratories, The University of Arizona, Tucson, AZ 85721, USA

## Abstract

**Background:**

Chromium is a toxic heavy metal, which primarily exists in two inorganic forms, Cr(VI) and Cr(III). Chromate [Cr(VI)] is carcinogenic, mutational, and teratogenic due to its strong oxidizing nature. Biotransformation of Cr(VI) to less-toxic Cr(III) by chromate-resistant and reducing bacteria has offered an ecological and economical option for chromate detoxification and bioremediation. However, knowledge of the genetic determinants for chromate resistance and reduction has been limited so far. Our main aim was to investigate chromate resistance and reduction by *Bacillus cereus *SJ1, and to further study the underlying mechanisms at the molecular level using the obtained genome sequence.

**Results:**

*Bacillus cereus *SJ1 isolated from chromium-contaminated wastewater of a metal electroplating factory displayed high Cr(VI) resistance with a minimal inhibitory concentration (MIC) of 30 mM when induced with Cr(VI). A complete bacterial reduction of 1 mM Cr(VI) was achieved within 57 h. By genome sequence analysis, a putative chromate transport operon, *chrIA*1, and two additional *chrA *genes encoding putative chromate transporters that likely confer chromate resistance were identified. Furthermore, we also found an azoreductase gene *azoR *and four nitroreductase genes *nitR *possibly involved in chromate reduction. Using reverse transcription PCR (RT-PCR) technology, it was shown that expression of adjacent genes *chrA*1 and *chrI *was induced in response to Cr(VI) but expression of the other two chromate transporter genes *chrA*2 and *chrA*3 was constitutive. In contrast, chromate reduction was constitutive in both phenotypic and gene expression analyses. The presence of a resolvase gene upstream of *chrIA*1, an arsenic resistance operon and a gene encoding Tn7-like transposition proteins ABBCCCD downstream of *chrIA*1 in *B. cereus *SJ1 implied the possibility of recent horizontal gene transfer.

**Conclusion:**

Our results indicate that expression of the chromate transporter gene *chrA*1 was inducible by Cr(VI) and most likely regulated by the putative transcriptional regulator ChrI. The bacterial Cr(VI)-resistant level was also inducible. The presence of an adjacent arsenic resistance gene cluster nearby the *chrIA*1 suggested that strong selective pressure by chromium and arsenic could cause bacterial horizontal gene transfer. Such events may favor the survival and increase the resistance level of *B. cereus *SJ1.

## Background

The wide use of chromium (Cr) in textile, leather tanning and electroplating industries with subsequent sewage disposal causes severe contamination of global soil-water systems [1,2]. Highly soluble, hexavalent chromium [chromate, CrO_4_^2-^] is very toxic. As an analogue of sulfate, chromate can enter bacterial and mammalian cells readily via sulfate transport systems [3]. The subsequent reduction of Cr(VI) by glutathione, thiols and other metabolites, and coproduction of reactive oxygen species (ROS) that damage DNA and other cellular components are the cause of the carcinogenic, mutational, and teratogenic potential of chromate [4-6]. On the other hand, the trivalent chromium [Cr(III)] is less bioavailable, thermodynamically stable and less toxic [7]. Accordingly, the reduction of toxic Cr(VI) to stable Cr(III) is an efficient way to remove chromate from soil and water systems.

Bioremediation of chromate-contaminated sites, especially when stimulating indigenous microbial communities, is getting more and more attention because of its economical and environmental friendly aspects compared to chemical and physical methods [8-10]. An increasing number of Cr(VI)- reducing bacteria have been detected and studied including a *pseudomonad *strain CRB5 [4], *Brucella *sp. [11], *Bacillus *sp. strain QC1-2 [12], *Burkholderia cepacia *MCMB-821 [13] and *Thermus scotoductus *strain SA-01 [14].

Bacteria have developed different strategies of chromate resistance including chromate efflux and chromate reduction. Efflux of chromate, which is mediated by the chromate transporter protein ChrA, has been confirmed in *Pseudomonas aeruginosa *[15,16], *Ochrobactrum tritici *5bvl1 [17] and *Shewanella *sp. ANA-3 [18]. Prior studies have not identified a chromate-responsive regulatory protein. Most chromate reduction studies have focused on soluble enzymes encoded by genes located on chromosomes [19]. However, very few of the proteins responsible for chromate reduction have been purified and characterized because of technical difficulties. When examining induction of chromate resistance and reduction genes, several strains including *Shewanella oneidensis *MR-1 [20], *Ochrobactrum tritici *5bvl1 [17] and *Ralstonia metallidurans *strain CH34 [21] have been shown to contain genes induced by chromate.

In this study, a chromate-resistant and reducing strain *Bacillus cereus *SJ1 was successfully isolated from chromium contaminated wastewater of a metal electroplating factory. Three chromate transporter related genes *chrA*, a chromate responsive regulator *chrI*, four *nitR *genes encoding nitroreductase and one azoreductase gene *azoR *possibly involved in chromate reduction were identified by the draft genome sequence. Using RT-PCR technology, we found that all of the five genes encoding putative chromate reductases appeared to be expressed constitutively. In contrast, the gene *chrA*1 encoding a transporter with high homology to other transporters linked to chromate resistance was up-regulated by the addition of Cr(VI) together with the adjacent putative transcriptional regulator *chrI*. Since *chrA*1 is probably regulated by *chrI*, this suggests identification of the first known chromate-responsive regulator.

## Results

### Identification of Cr(VI)-reducing *B. cereus *SJ1 that is highly chromate resistant

Strain SJ1 showing both high Cr(VI) resistance and reduction abilities was isolated from industrial wastewater of a metal plating factory. SJ1 was a Gram positive, rod shaped bacterium. The 16 S rDNA sequence was used for bacterial identification. SJ1 showed the highest identity (100%) with *B. cereus *03BB102 [GenBank: CP001407] and was hereafter referred to as *B. cereus *SJ1.

**Figure 1:**
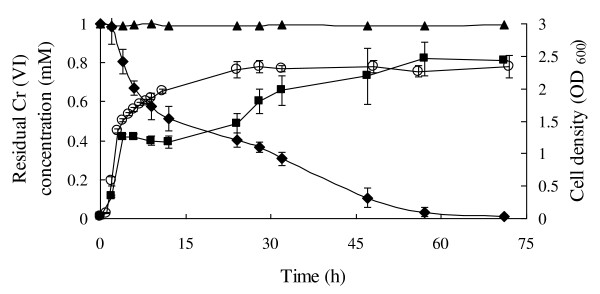
**Chromate reduction and growth curves of *B. cereus *SJ1**. *B. cereus *SJ1 growth curves in LB medium with (■) and without (○) 1 mM K_2_CrO_4_. (♦), Cr(VI) reduction of *B. cereus *SJ1 in LB medium (pH 7.0) with 1 mM K_2_CrO_4_. (▲), LB medium (pH 7.0) amended with 1 mM K_2_CrO_4 _without bacterial inoculation as a control. Error bars represent standard deviation of triplicate samples.

**Figure 2:**
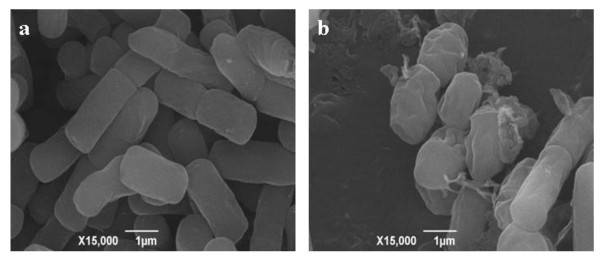
**SEM micrographs of *B. cereus *SJ1 cells**. (a), *B. cereus *SJ1 cells grown in LB medium for 24 h without K_2_CrO_4_; (b), *B. cereus *SJ1 cells grown in LB medium amended with 1 mM K_2_CrO_4 _for 24 h. Scale bars: 1 μm.

*B. cereus *SJ1 showed rapid reduction of Cr(VI) aerobically. Cell growth and Cr(VI) reduction by *B. cereus *SJ1 were monitored spectrophotometrically (Figure 1). The growth rate of SJ1 was rapid. It reached log-phase in 4-6 h in LB medium and the growth rate was decreased by addition of 1 mM chromate. In the first 12 h, the chromate reduction rate was shown to be fastest under optimum pH (7.0) and temperature (37°C) conditions (data not shown). After 57 h of incubation, up to 97% soluble Cr(VI) was reduced and white precipitate was visible at the bottom of the flasks [22]. Abiotic Cr(VI) reduction was not observed in cell-free LB medium (Figure 1). After cultivation of *B. cereus *SJ1 for 24 h with 1 mM K_2_CrO_4_, the cell became shorter with roughness on the cell surfaces possibable caused by the environmental stress of chromate (Figure 2)

### General features of *B. cereus *SJ1 draft genome and genes involved in chromate metabolism

Draft genome sequence analysis of *B. cereus *SJ1 showed a genome size of about 5.2 Mb distributed in 268 contigs with an average GC content of 35.4%, containing 5,708 putative coding sequences (CDSs). There are 100 tRNA genes representing all 20 amino acids and 6 scattered ribosomal RNA genes identified on the draft genome. The likely origin of replication of the chromosome of *B. cereus *SJ1 was located in a 9.0 kb region that included co-localization of six genes (*rpmH, gyrA, gyrB, recF, dnaN *and *dnaA*). It was localized by comparing its draft genome to complete genomes of several strains of the *B. cereus *group though MUMmer3.20.

**Figure 3:**
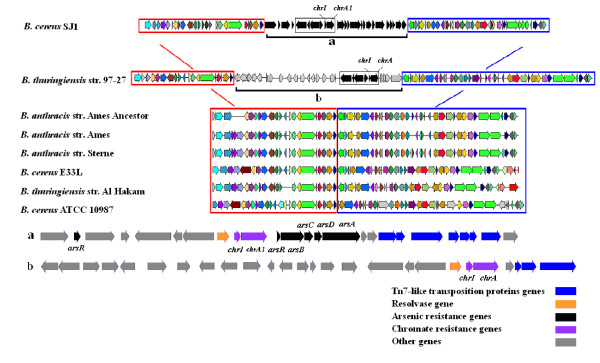
**Comparison of genetic determinants of chromate resistance in other bacterial strains versus *B. cereus *SJ1**. (a) Genetic context of the chromate operon *chrIA *and arsenic resistance operon *arsRBCDA *in *B. cereus *SJ1. (b) Genetic context of the chromate operon *chrIA*1 in *B. thuringiensis *serovar konkukian str. 97-27. *B. thuringiensis *str. 97-27 [GenBank: AE017355]; *B. anthracis *str. Ames Ancestor [GenBank: AE017334]; *B. anthracis *str. Ames [GenBank: NC003997]; *B. anthracis *str. Sterne [GenBank: AE017225]; *B. cereus *E33L [GenBank: CP000001]; *B. thuringiensis *str. Al Hakam [GenBank: NC008600] and *B. cereus *ATCC 10987 [GenBank: AE017194].

Three putative chromate transporter genes, *chrA*1, *chrA*2 and *chrA*3 were identified in the genome of *B. cereus *SJ1 (Additional file 1). The *chrA*1 encoding ChrA protein showed the highest amino acid identity (97%) with a homologous protein annotated as chromate transporter in *Bacillus thuringiensis *serovar konkukian str. 97-27 [GenBank: YP036530]. Interestingly, *chrA*1 gene (locus_tag: BCSJ1_04594, 1,194 bp) located downstream of a potential transcriptional regulator gene *chrI *(locus_tag: BCSJ1_04599, 309 bp). The region of *chrA*1 and *chrI *also contained several CDSs encoding homologs of Tn7-like transposition proteins and a resolvase that could potentially have been involved in horizontal gene transfer events (Figure 3a). This region covered 26 kb sequence and showed lower GC content (32.8%) compared with the average GC content of *B. cereus *SJ1's whole genome (35.4%). A similar region was also observed in *B. thuringiensis *serovar konkukian str. 97-27 (Figure 3b), but was absent in other *B. cereus *genomes. Remarkably, differing from *B. thuringiensis *serovar konkukian str. 97-27, this region of *B. cereus *SJ1 contained several genes related to arsenic resistance including genes encoding an arsenic resistance operon repressor ArsR, arsenic resistance protein ArsB, arsenate reductase ArsC, arsenic chaperon ArsD and arsenic pump ATPase ArsA (Figure 3a). This may indicate a very recent horizontal gene transfer (HGT) event since genes located upstream of *chrIA*1 and downstream of arsenic resistance genes were resolvase and Tn7-like transposition protein ABBCCCD in both strains. Furthermore, four *nitR *genes encoding nitroreductases and a gene *azoR*, encoding an azoreductase that had previously been reported to catalyze chromate reduction [19,23] were found in the draft genome (Additional file 1, 99-100% amino acid identities).

### Heavy metal tolerance of *B. cereus *SJ1 and putative genes responsible for heavy metal resistance

Since *B. cereus *SJ1 was isolated from industrial wastewater containing various toxic elements in addition to chromium, the MICs of *B. cereus *SJ1 for these heavy metals were determined. For *B. cereus *SJ1, the highest resistance was found for As(V), while Hg(II) was the most toxic compared to the other metal ions. When *B. cereus *SJ1 was incubated with increasing As concentration, no viable cells were recovered at concentrations above 50 mM As(V) and 4 mM As(III). The MICs of *B. cereus *SJ1 for Cu(II), Co(II), Ni(II), Cd(II), Ag(I) and Hg(II) were 0.9 mM, 0.8 mM, 0.7 mM, 0.2 mM, 0.02 mM and 0.007 mM, respectively. In order to survive in such unfavorable habitat, *B. cereus *SJ1 must have various determinants to tolerate such harsh conditions. For example, the copper concentration of the wastewater was as high as 0.65 mM and the MIC of *B. cereus *SJ1 to copper was 0.9 mM in R2A medium. When we analyzed the genome sequence of *B. cereus *SJ1, several genes related to copper resistance including copper-exporting P-type ATPase CopA, copper export protein CopC, copper resistance protein CopD, copper homeostasis protein CutC and two multicopper oxidases were identified. Furthermore, many other putative heavy metal resistance genes including those for As, Zn, Mn, Co, Cd, Te and Hg were also identified in the *B. cereus *SJ1 draft genome (Additional file 2).

### Chromate reduction is constitutive

**Figure 4:**
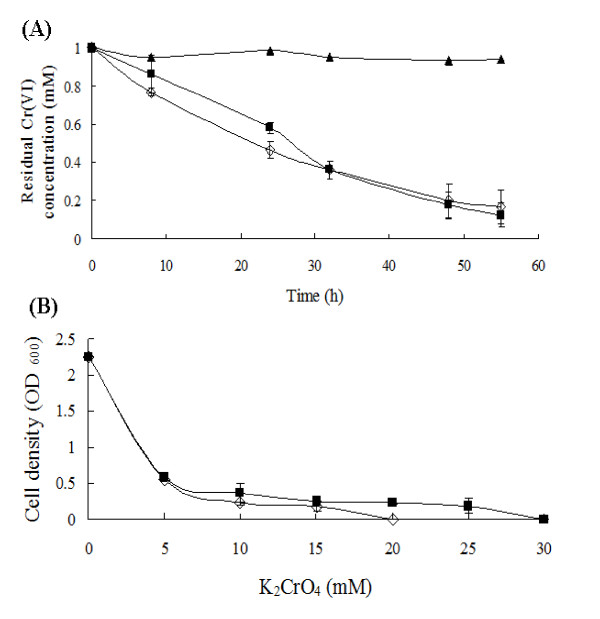
**Chromate resistance and reduction of *B. cereus *SJ1**. Chromate reduction **(A) **and resistance **(B) **analysis of *B. cereus *SJ1 uninduced (◊) and induced with (■) 1 mM K_2_CrO_4 _for 8 h before bacterial inoculation in LB medium (pH 7.0). *B. cereus *SJ1 was incubated for 48 h before growth was measured for Cr resistance determination. (▲), amended with 1 mM K_2_CrO_4 _without bacterial inoculation as a control. Error bars represent standard deviation of triplicate samples.

**Figure 5:**
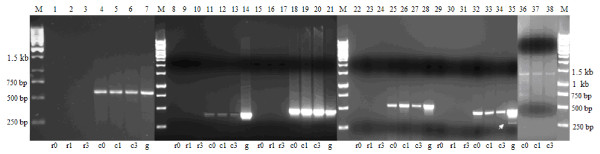
**RT-PCR analysis of putative chromate reduction genes *nitR *and *azoR***. M, 1 kb DNA ladder. r, negative control for RT, obtained using total RNA (after DNase I treatment) as the template for PCR amplification, to verify that no genomic contamination was present in the RNA extract; c, RT-PCR product using the first strand cDNA as the template; g, PCR positive control obtained using genomic DNA from *B. cereus *SJ1 as the template. 0, 1 and 3 after r and c represent samples uninduced and induced by 0.3 mM K_2_CrO_4 _for 1 h and 3 h, respectively. Lanes 1-7, *nitR*1 (locus_tag: BCSJ1_00500, 592 bp); Lanes 8-14, *azoR *(locus_tag: BCSJ1_06081, 413 bp); Lanes 15-21, *nitR*2 (locus_tag: BCSJ1_14230, 480 bp); Lanes 22-28, *nitR*3 (locus_tag: BCSJ1_17540, 546 bp); Lanes 29-35, *nitR*4 (locus_tag: BCSJ1_02410, 477 bp); Lanes 36-38, RT-PCR of 16 S rRNA genes. The arrow indicates a non-specific band.

The difference in chromate reducing ability of *B. cereus *SJ1 with and without Cr(VI) induction was not significant (Figure 4A). Although less rapid chromate reduction was observed in *B. cereus *SJ1 cells induced before inoculation during the first 32 h, both cultures emerged at approximately 85% chromate reduced within 55 h. No abiotic Cr(VI) reduction was observed in LB medium without bacterial inoculation. Induction of genes possibly responsible for chromate reduction was also evaluated by RT-PCR. As shown in Figure 5, all the four *nitR *genes and the *azoR *gene were expressed constitutively. The results of RT-PCR were in agreement with the bacterial chromate induction experiment (Figure 4A), strongly indicating no significant regulatory influence of chromate on chromate reduction.

### Expression of *chrA*1 is inducible by chromate

Using the procedure described in Methods, we found that the uninduced and induced cells grew to similar cell densities in medium containing 5 mM Cr(VI) as determined spectrophotometrically at OD_600_. However, the induced cells grew to higher cell densities than the uninduced cells at higher Cr(VI) concentrations in the growth medium. The MIC of induced *B. cereus *SJ1 to K_2_CrO_4 _was 30 mM whereas that of the uninduced strain was 20 mM (Figure 4B).

**Figure 6:**
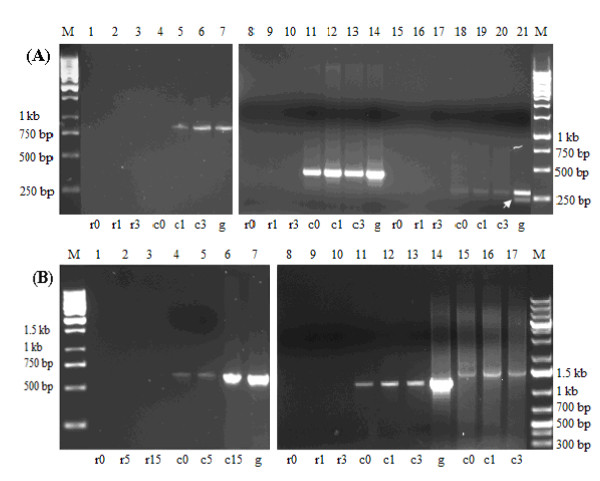
**RT-PCR analysis of *chrA, chrI *induction and *chrI-chrA*1 co-transcription**. The M, r, c, g were identical to these of Figure 5. **(A)**, RT-PCR analysis of expression of *chrA*'s. Lanes 1-7, chromate resistance gene *chrA*1 (locus_tag: BCSJ1_04594, 946 bp); Lanes 8-14, *chrA*2 (locus_tag: BCSJ1_18833, 491 bp); Lanes 15-21, *chrA*3 (locus_tag: BCSJ1_18828, 354 bp). **(B)**, RT-PCR analysis of *chrI *induction and *chrI-chrA*1 co-transcription. 5 and 15 after r and c represent samples induced by 0.3 mM K_2_CrO_4 _for 5 min and 15 min, respectively. Lanes 1-7, transcriptional regulator gene *chrI *(locus_tag: BCSJ1_04599, 604 bp); Lanes 8-14, *chrI-chrA*1 (1,130 bp). Lanes 15-17, RT-PCR of 16 S rRNA genes. The arrow indicates a non-specific band.

Induction of the different *chrA *genes was also evaluated by RT-PCR using RNA isolated from cultures grown in the presence and absence of 0.3 mM Cr(VI) from 0 h to 3 h (Figure 6A). A *chrA*1-specific fragment was clearly visible when Cr(VI) was added that was absent when no Cr(VI) was added (Lane 4 vs 5 and 6), indicating expression of *chrA*1 was induced by the addition of Cr(VI). In contrast, RT-PCR of the other two *chrA *genes, *chrA*2 and *chrA*3, showed that both were expressed constitutively. No products were found using total RNA as the template for PCR amplification, thus indicating the absence of DNA contamination in the total RNA preparations.

### *chrI*, encoding a transcriptional regulator, is regulated by chromate

The *chrI *gene located upstream of *chrA*1 encodes a protein with 98% amino acid sequence identity to the PadR-family transcriptional regulator from *B. thuringiensis *serovar konkukian str. 97-27 [GenBank: YP036529]. As *chrI *was a potential transcriptional regurator, it should be responsive to the inducer (Cr), so we analyzed the transcription of *chrI *at 5 and 15 min after addition of K_2_CrO_4_. A very weak PCR product was detected with cDNA from uninduced cells as shown in Figure 6B. The level of the *chrI *gene transcript was 16-fold higher (analyzed using BandScan 5.0 program) in cells induced for 15 min compared to the uninduced culture (lane 4 vs 6), confirming substrate-mediated regulation of *chrI*. To confirm the hypothesis that *chrI-chrA*1 was transcribed as a single transcription unit, RT-PCR was carried out with mRNA prepared from *B. cereus *SJ1 grown with and without K_2_CrO_4 _(0.3 mM) as described above. PCR products of the expected size (1,130 bp) were obtained with cDNA from both induced and uninduced cultures as the templates (Figure 6B), which indicated *chrI *and *chrA*1 were arranged as an operon. No PCR products were amplified using total RNA as the template that was designed to detect DNA contamination.

The arrangement of *chrI *genes in an operon together with *chrA *encoding a chromate transporter can be detected in both Gram positive and Gram negative bacteria (Additional file 3). An alignment of ChrI homologs was constructed using ChrI of *B. cereus *SJ1 and other related proteins encoded in operons having a *chrI *gene adjacent to a *chrA *gene (Additional file 4). The more-conserved domains were located in the N- and C-terminal regions. Within the conserved domains, two amino acids, lysine and arginine, were identified that might be involved in chromate binding and recognition.

## Discussion

Chromate-reducing bacteria have been discovered in both contaminated and non-polluted environments [1,13,24,25]. In this study, a chromate-resistant strain *B. cereus *SJ1 was isolated from chromium contaminated wastewater of a metal plating factory in China. *B. cereus *SJ1 showed a rapid growth rate in chromate containing medium and efficient chromate-reducing ability under aerobic conditions. Since the isolation site for *B. cereus *SJ1 was contaminated with as much as 1.89 mg Cr per liter (36.28 μM), we reasoned that genes conferring chromate resistance could be present in this strain. By genome sequencing analysis, we were able to identify a putative chromate transport operon *chrIA*1 and two additional *chrA *genes encoding putative chromate transporters that confer chromate resistance. Furthermore, we also found an azoreductase gene *azoR *and four *nitR *genes that encode nitroreductases which may catalyze reduction of chromate [19,23].

The membrane transporter protein ChrA has been shown to be responsible for extrusion of chromate ions across the cytoplasmic membrane in *P. aeruginosa *[15,16], *Ochrobactrum tritici *5bvl1 [17] and *Shewanella *sp. ANA3 [18]. It was demonstrated that the chromate transporter ChrA functions as a chemiosmotic pump that extrudes chromate using proton-motive force [15]. ChrA protein belongs to the CHR superfamily which includes dozens of putative homologs from all three domains of life [26]. Cr(VI) induction of *B. cereus *SJ1 in this study conferred the ability to survive at a higher chromate concentration. Exposure to chromate resulted in the up-regulation of *chrA*1 and higher chromate resistance. Possibly increased level of ChrA1 is responsible for higher chromate resistance.

The *chrI *gene product located upstream of *chrA*1 showed a high homology to PadR-family transcriptional regulators. The *padA *gene encoding phenolic acid decarboxylase, is a member of the PadR family that has been identified as a transcriptional repressor in *Pediococcus pentosaceus *[27] and *Lactobacillus plantarum *[28]. Although genes encoding PadR homologs located either upstream or downstream of putative chromate transporter gene *chrA *have been identified in many genera, such as *B. thuringiensis *serovar konkukian str. 97-27 [GenBank: YP036529], *Oceanobacillus iheyensis *HTE831 [GenBank: NP694199], *B. licheniformis *ATCC 14580 [GenBank: YP093604) and *Alkaliphilus oremlandii *OhILAs [GenBank: YP001512811], the real function of a PadR homolog associated with chromate resistance has never been reported. In this study, this gene encoding a PadR homolog was renamed as *chrI *since it was induced by chromate. By an alignment of most PadR-like regulators which form an operon with the chromate transporter gene *chrA*, highly conserved basic amino acids (lysine and arginine) were identified in ChrI and the homologs that might be involved in chromate binding and recognition because they would carry a positive charge under physiological conditions. Possibly the negatively charged oxyanion CrO_4_^2- ^would preferentially bind the basic, positively charged amino acids conserved in the putative transcriptional regulator ChrI.

A strong selective pressure for transformation of metal- and metalloid-related resistance genes is present in heavy metal contaminated environments [29,30]. Horizontal gene transfer (HGT) events driven by mobile genetic elements, such as phages, plasmids, insertion sequences, integrons and transposons, have been shown to provide microbes with a wide variety of adaptive traits for microbial survival under hostile environmental conditions. In this study, *B. cereus *SJ1 was isolated from wastewater contaminated with multiple heavy metals. The presence of a resolvase gene upstream of *chrIA*1 and an arsenic resistance gene cluster, and the Tn7-like transposition proteins ABBCCCD gene downstream of the arsenic resistance operon in *B. cereus *SJ1 but absent in other strains of *B. cereus *implied the possibility of a recent HGT event. Interestingly, other strains of *B. cereus *harbor a gene encoding CHRD-domain-containing protein adjacent to the *chrA *gene. Whether these proteins have a regulatory role is currently unknown [31]. In addition, ChrA1 from *B. cereus *SJ1 is only distantly related to ChrA proteins from other strains of *B. cereus *indicating potential horizontal gene transfer from other Gram-positive bacteria as an adaptation to survive in a highly chromate contaminated environment.

Chromate can be reduced nonenzymatically as well as by various bacterial enzymes. Dihydrolipoamide dehydrogenase from *Thermus scotoductus *SA-01 [32], azoreductase in *Shewanella oneidensis *[19] and flavoproteins from *P. putida *and *E. coli *[3] were previously reported to be associated with Cr(VI) reduction. Compared to the one electron transfer chromate reductase gene *chrR *from *P. putida, yieF *from *E. coli *was proposed to be a more appropriate gene for bioremediation applications because of the three-electron transfer ability of its gene product and consequently, the generation of fewer reactive oxygen species (ROS) [33]. In our study, one azoreductase gene *azoR *and four *nitR *genes encoding nitroreductase obtained from *B. cereus *SJ1 showed high identities with other Cr(VI) reductases and were expressed constitutively. Since Cr(VI) reduction of strain SJ1 was not inducible by chromate, other potential chromate reductases in *B. cereus *SJ1 must also be constitutively expressed and the enzyme activity is probably adventitious.

## Conclusion

This study describes insights into the chromate resistance and reduction capabilities of *B. cereus *SJ1 using both physiological and molecular techniques. The expression of the chromate transporter gene *chrA1 *was inducible by Cr(VI) and most likely regulated by *chrI*. Even though the physiological function of ChrI has not been verified due to the absence of a genetic system for this Gram positive strain, ChrI is most likely the first identified chromate responsive regulator. In addition, genome analysis identified a number of putative genes encoding gene products with possible functions in chromate resistance and reduction which may be the basis for the observed high chromate resistance and reduction ability of this strain. Furthermore, possible horizontal gene transfer events indicated in this study may have enabled *B. cereus *SJ1 to survive in metal (loid) contaminated environments.

## Methods

### Isolation of Cr(VI)-resistant and reducing bacteria

Industrial wastewater samples were obtained from a metal electroplating factory in Guangdong, China. The total concentrations of Cr, Cu, Zn, Mn, Pb, Co, As and Cd in this sample determined by atomic absorption spectrometry were 36.28 μM, 0.65 mM, 24.88 μM, 7.83 μM, 0.49 μM, 0.41 μM, 0.32 μM, and 0.007 μM, respectively. Isolation of chromate-resistant and reducing bacteria was performed as described [34]. The abilities of the chromate-resistant bacteria to reduce Cr(VI) (K_2_CrO_4_) were determined using a spectrophotometric method using the reagent 1, 5-diphenylcarbazide (DPC) [34]. Several chromate-resistant bacteria were isolated and strain SJ1 was chosen for this study. The 16 S rDNA of strain SJ1 was obtained from the genome sequence (see below) and analyzed by BlastN searching tools http://www.ncbi.nlm.nih.gov/blast. Cell morphologies were examined under a scanning electron microscope (SEM; JSM-6390, JEOL, Japan) with 20,000 V accelerating voltage and 15,000 times enlargement.

### Determination of the minimal inhibitory concentrations (MICs) of heavy and transition metals and metalloids

The MIC, defined as the lowest concentration of heavy metals that inhibited growth in R2A broth (Becton Dickinson, MD, USA), was performed with strain SJ1. A 1% inoculum of an overnight culture was introduced into R2A medium amended with different concentrations of CuCl_2_, NiCl_2_, Co(NO_3_)_2_, Na_2_HAsO_4_, NaAsO_2_, HgCl_2_, CdCl_2 _and AgNO_3_, incubated at 37°C on a rotary shaker at 200 rpm for 3 days. MIC values were determined spectrophotometrically at OD_600_.

### Chromate resistance and reduction assays

The exponential phase cultures of uninduced, and induced with 1 mM K_2_Cr_2_O_6 _for 8 h, were adjusted to the same OD_600_. One hundred microliters of each culture was added to 10 ml fresh LB medium with increasing amounts of K_2_CrO_4, _and incubated at 37°C with 200 rpm shaking for 3 days. The OD_600 _values were then determined spectrophotometrically. For chromate reduction, the uninduced and induced cultures were prepared as above and inoculated into 100 ml LB medium amended with 1 mM K_2_CrO_4 _and incubated at 37°C on a rotary shaker at 200 rpm for about 60 h. The residual Cr(VI) concentration was monitored as described above. LB medium with 1 mM K_2_CrO_4 _without bacterial cells was incubated as a negative control to monitor abiotic chromate reduction.

### Sequencing of the *B. cereus *SJ1 genome

High-molecular-mass genomic DNA isolated from *B. cereus *SJ1 using Blood & Cell Culture DNA Mini Kit (Qiagen, MD, USA) was used to construct a 4 kb to 40 kb random genomic library. Whole genome shotgun sequencing was performed by the University of Arizona Genetics Core facility, using a Roche 454 Genome Sequencer FLX instrument. The *B. cereus *SJ1 DNA sample was loaded onto one region of a standard four-region plate. A local Linux computing cluster was used for signal processing on the images produced by the FLX instrument. The Roche gsassembler software version 2.0.01 was used for de novo assembly of the 271,408 reads. Using the default assembly parameters, 141 contigs of length greater than 500 bp were built, along with 127 shorter contigs. These 268 contigs were submitted to the RAST annotation server [35] for subsystem classification and functional annotation. Genome comparison was performed through SEED server http://rast.nmpdr.org/seedviewer.cgi and the subsequent results were modified manually. GC content was analyzed using CLC Main Workbench 5 program http://www.clcbio.com. The NCBI Prokaryotic Genomes Automatic Annotation Pipeline was used for gene annotation in preparation for data submission to GenBank. http://www.ncbi.nlm.nih.gov/genomes/static/Pipeline.html.

### Gene expression and co-transcription analyses

RT-PCR was used to assess induced expression and co-transcription of the chromate resistance and reduction related genes of strain SJ1. Total RNA was obtained from mid-exponential phase strain SJ1 cells grown from 0 h to 3 h in the presence or absence of 0.3 mM K_2_CrO_4 _in LB medium. Total RNA was isolated by the RNeasy Mini Kit (Qiagen) and then digested with DNase I (Fermentas, MD, USA) to remove any DNA. The OD_260 _values were then determined spectrophotometrically for the total RNA concentration. Equal amounts of total RNA were used to perform cDNA synthesis using iScript™Select cDNA Synthesis Kit (Biorad, CA, USA). Standard PCR programs were used to generate amplicons from 3 μl of the reverse transcription reaction mixture using the specific primer pairs listed in Additional file 5. PCR amplification using RNA as template was served as the control to investigate the potential presence of DNA contamination. The relative levels of the cDNAs of RT-PCR were determined by densitometric analyses using BandScan 5.0 software (GLyko Inc., Novato, CA, USA) using 16 S rRNA genes as references.

### Deposition of strain and nucleotide sequences

*B. cereus *SJ1 was deposited in The Agricultural Research Service Culture Collection, USA (NRRL http://nrrl.ncaur.usda.gov) under the accession number of NRRL B-59452. The Whole Genome Shotgun project has been deposited at DDBJ/EMBL/GenBank http://www.ncbi.nlm.nih.gov/sites/genome under the accession number of ADFM00000000. The version described in this paper is the first version, ADFM01000000.

## Authors' contributions

All authors participated in the design of the study and data analyses. MH carried out bacterial isolation, resistant and reduction assay, molecular genetic studies and manuscript preparation. XL carried out the genome analysis. SM carried out genomic sequencing and the whole genome shotgun submission. LG performed the electron microscope analysis. CR participated in the design of the experiments and helped to draft the manuscript. GW is the principal investigator of the funded project. She coordinated the study and helped to draft the manuscript. All authors read and approved the final manuscript.
